# Comparative analysis of stirred catalytic basket bio-reactor for the production of bio-ethanol using free and immobilized *Saccharomyces cerevisiae* cells

**DOI:** 10.1186/s13568-017-0460-8

**Published:** 2017-07-28

**Authors:** Amir Hussain, Martin Kangwa, Marcelo Fernandez-Lahore

**Affiliations:** Downstream Bioprocessing Laboratory, Department of Life Sciences & Chemistry, Jacobs University Bremen, Campus Ring 1, 28759 Bremen, Germany

**Keywords:** Stirred-Catalytic-Basket-BioReactor, *Saccharomyces cerevisiae*, Sponge, Alginate

## Abstract

The successful industrial production of ethanol and fine chemicals requires the development of new biocatalytic reactors and support materials to achieve economically viable processes. In this work, a Stirred-Catalytic-Basket-BioReactor using various immobilizing foams as support material and compared to free cells were used, focusing mainly on; (i) effect of mass-transfer on cells physiology and (ii) ethanol productivity. The performance of the reactor was further evaluated by ethanol volumetric productivity, yield and time for process completion and it was found that the variation of ethanol production and diffusion of the substrate in fermentation process are co-related with the stirrer speed and initial glucose concentration. It was also observed that the time difference for glucose consumption between free and immobilized cells (alginate and sponges) tends to increase by increasing the glucose concentration in the medium. We found that at higher stirrer speed (500 rpm) when using higher glucose concentration (200 g/l), ethanol volumetric productivity increased significantly in the sponge (85 g/l) as compared to alginate beads (79 g/l) and free cells (60 g/l). From the data obtained, it can be concluded that sponges are the best support material for attaining higher ethanol productivity. A stirred catalytic basket bioreactor with yeast cells immobilized in polyethylene sponge gives higher ethanol production at a higher glucose consumption rate, and this productivity is due to higher mixing efficiency and reduced external as well as internal mass transfer limitations. The potentials of the reactor rank it as a remarkable ethanol/fine-chemical production approach that needs further investigations.

## Introduction

Air-pollution currently witnessed globally, caused mainly by the extensive usage of fossil fuel has brought about devastating effects both environmentally and health, thereby encouraging extensive scientific research in finding alternative and cheaper bio-fuel like ethanol via microbial fermentation in the bioreactor. The traditional setups used in the ethanol production like membrane bioreactor, airlift bioreactor, fixed bed bioreactors and stirred tank reactors have some drawbacks of less product yield due to low mass and heat transfer, inefficient conversion of substrate, uneven mixing and shear stress on biocatalysts (Hussain et al. 2015a). To overcome these problems and to improve the efficiency of a bioreactor, four most important factors need to be put in consideration i.e. choice of the fermentation process, biocatalyst, support for immobilization and bioreactor design.

**Fig. 1:**
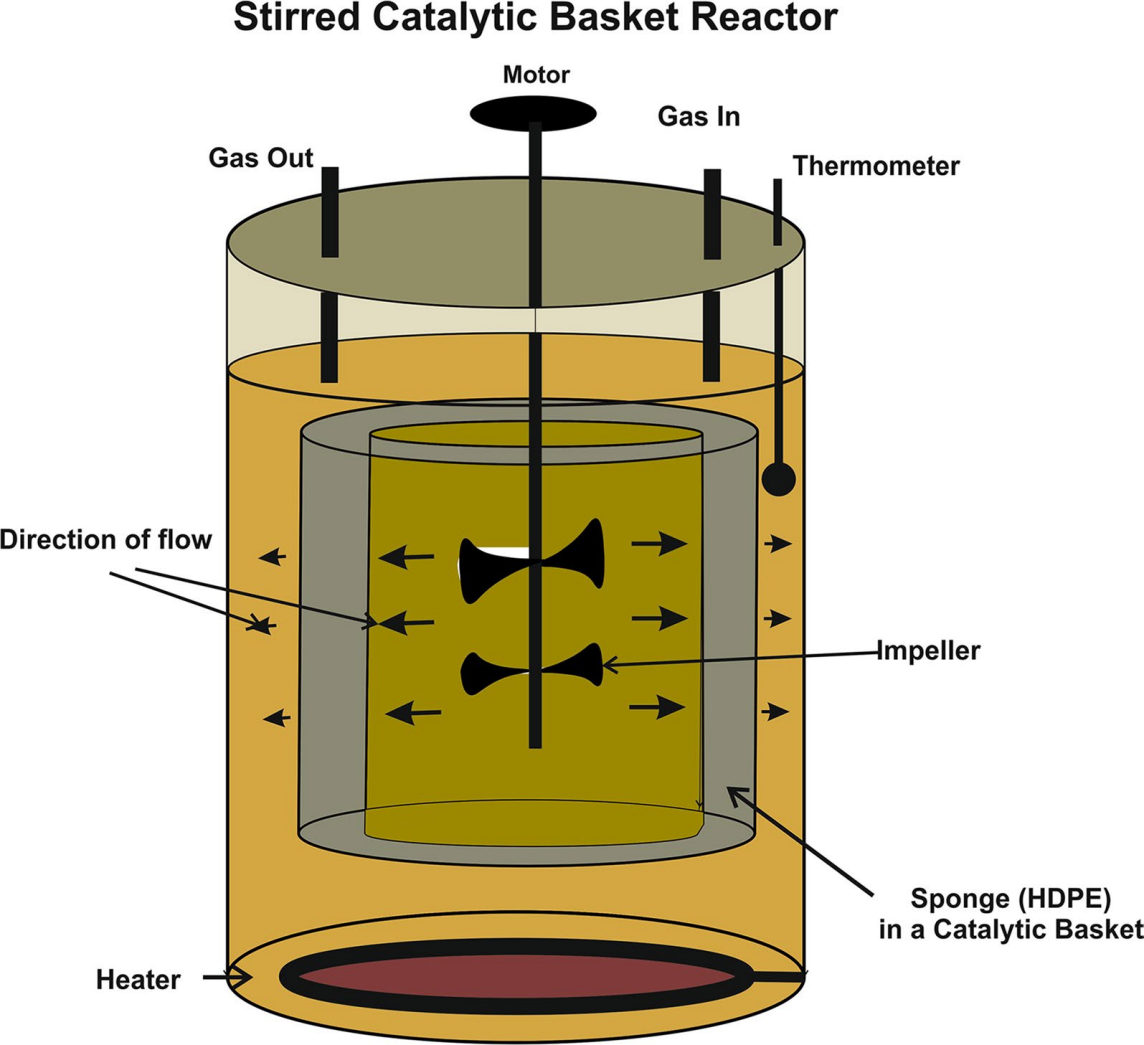
Schematic representation of a Stirred Catalytic Basket Bioreactor

**Fig. 2:**
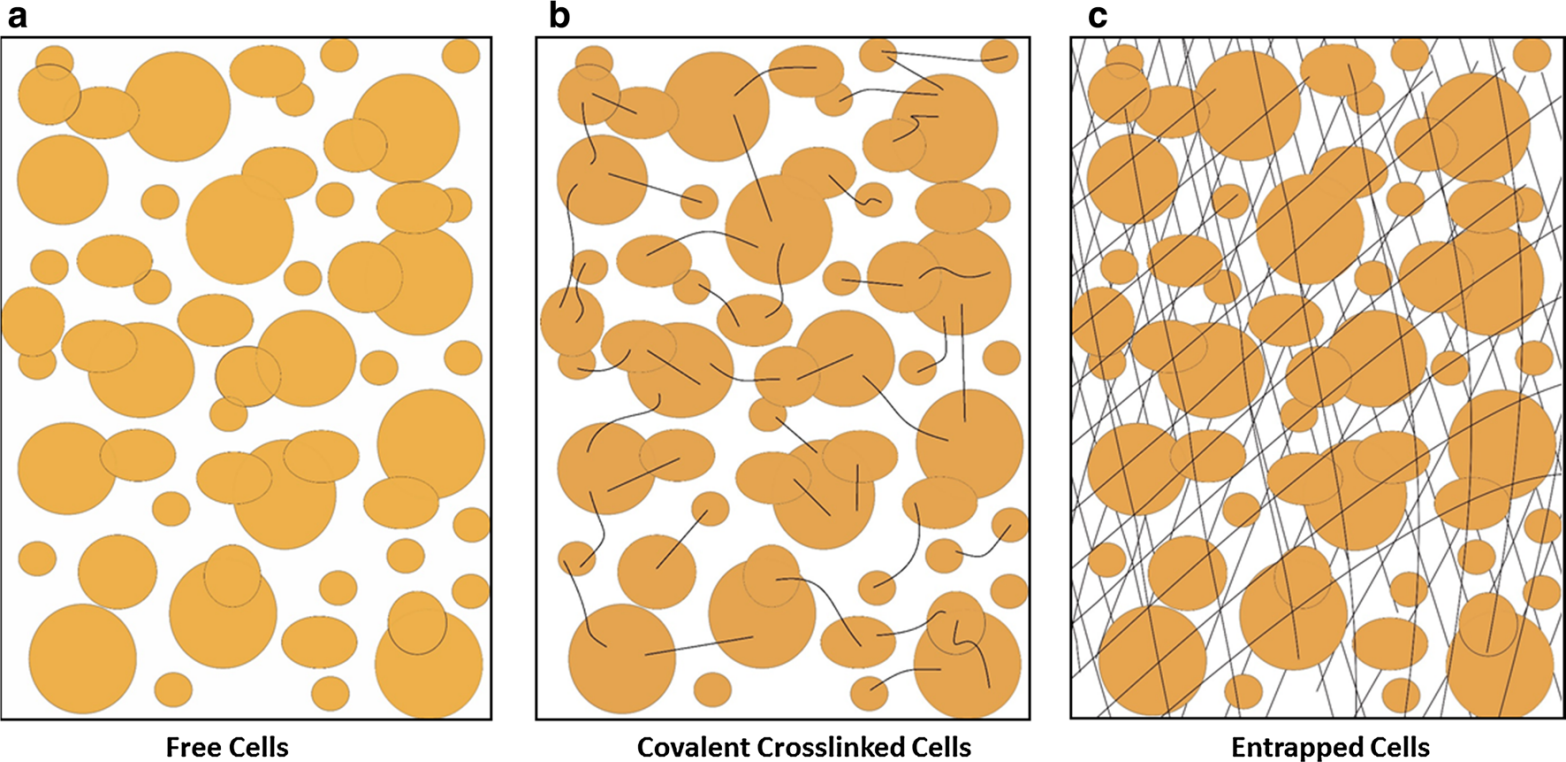
Schematic representation of cell immobilized techniques:** a** Free cells,** b** Covalent crosslinked,** c** Entrapped

There are three microbial fermentation processes currently used for ethanol production namely: batch, fed-batch and continuous. In this study, batch fermentation process was selected as it has a single fermentation cycle, thereby less operational time. During fermentation, biocatalysts like living cells such as *Saccharomyces cerevisiae* are utilized for the production of ethanol and can also be used for the production of a variety of fine chemicals and active pharmaceutical ingredients (Khor and Uzir 2011). In traditional ethanol fermentation technology, freely suspended yeast cells have been used in the batch. Moreover, several drawbacks like more fermentation time and low volumetric productivity were found in this technology, and this is due to continuous changes in the external physical factors and biological activity of yeast. To achieve optimal conditions for metabolic activity, yeast must maintain the intracellular physical and chemical parameters (Bauer and Pretorius 2000). Free cells are more sensitive to nutrient depletion, pH variations, and certain inhibitory compounds because these are directly exposed to the changing environment. Additional most important factor and aim of this study were the need to prepare support material for cell immobilization and further use in newly developed stirred catalytic basket bioreactor (SCBBR) (Fig. 1). Cell immobilization is defined as the localization of intact cells into a defined region of space with the preservation of catalytic activity. Currently, there are different immobilization methods available; its choice depends on the nature of the application of cells like; physical entrapment, attachment or adsorptions, self-aggregation by flocculation (Fig. 2). Physical entrapment means entrapment of cells within a porous polymeric matrix such as calcium alginate, carrageenan, chitosan and other polymer beads. The attachment or adsorption method involves the reversible attachment of biomass to a solid support mainly by electrostatic, ionic and hydrogen bonding interactions (Pilkington et al. 1998). This type of method is usually used for different carriers like DEAE cellulose, porous glass, sponges and wood blocks (Pilkington et al. 1998; Williams and Munnecke 1981).

The main objective was to observe the performance of SCBBR with immobilized cells in different matrixes and compare to free cells in STR-stirred tank reactor regarding; (i) effect of mass transfer on cells physiology and (ii) ethanol productivity and understand the mechanism of external and internal mass transfer effect on immobilized system in conjunction with the performance of SCBBR in ethanol production. The performance of SCBBR was evaluated by volumetric productivity, ethanol yield and time for process completion.

## Materials and methods

### Reactors

A stirred tank reactor-STR and SCBBR was bought from Bioengineering Inc, Germany. Spectrophotometer, GC column CP-WAX 58, Gas chromatography HP 5890 series II, where from Hewlett-Packard, Avondale, PA, USA. All other chemicals, including yeast extract nitrogen base without amino acid, ammonium acetate, amino acid mixture, sodium alginate, calcium chloride, sodium chloride, chitosan, hydrochloric acid, potassium sodium tartrate, dinitro salicylic acid (DNS), *n*-butanol, ethyl acetate, glucose, peptone, yeast extract, agar, sodium hydroxide were of analytical grades and purchased directly from Sigma (USA) and Applichem (Germany).

### Microorganism

The yeast strains *Saccharomyces cerevisiae* (baker yeast) was obtained from DHW Vital Gold, Nürnberg, Germany, while the *Saccharomyces cerevisiae* Ethanol Red11 strain was purchased from Fermentis Inc, Germany and were stored at 4 and −80 °C, respectively.

### Strain preservation

For the strain preservation, the yeast strain was initially prepared in a sterile cultivation media and 1.0 ml of a late log or early stationary phase culture solution was mixed with equal volume of 30% glycerol (w/v) solution into sterile 4 mL screw-cap vials mixed and freeze on dry ice, and store at −80 °C. For reviving, the strain was scraped and further streaked onto plates.

### Inoculum preparation

For the culture preparation, Ethanol Red 11 strain was refreshed by streaking onto YPD agar plate (1% Yeast extract, 2% Peptone and 2% Glucose, 2% agar), incubated for 2 days at 35 °C. The resulting single colonies were used to start a fresh culture. Twenty milliliters of YPD media (1% yeast extract, 2% Peptone and 10% d-Glucose) in a 100 ml flask was inoculated with a single colony of Yeast Ethanol Red 11 grown overnight at 35 °C with vigorous shaking at 250 rpm. One percent of the pre-culture was used to inoculate 2 l Erlenmeyer baffled flask containing 1000 ml YPD media final volume. The inoculated flask was incubated on a rotary shaker at 200 rpm and 35 °C for 24 h. Furthermore, the cells were collected by centrifugation at 4000 rpm for 15 min, washed twice with sterile distilled water, centrifuged and re-suspended in sterile water to obtain a dense cell suspension.

### Fermentation medium and cultivation

For this stage, minimal media was utilized in the cultivation process, prepared with 6.7 g/l yeast extract nitrogen base without amino Acid, 1.7 g/l ammonium acetate and glucose (4 and 10 g/l) were prepared separately and mixed after sterilizing (121 °C, 20 min.). These different amino acids were mixed to prepare “amino acid mixture” (100×); 200 mg l-arginine, 1000 mg l-aspartic acid, 1000 mg l-glutamic acid, 300 mg l-lysine, 500 mg l-phenylalanine, 4000 mg l-serine, 2000 mg l-threonine, 300 mg l-tyrosine, 1500 mg l-valine. All components were dissolved in distilled water by adjusting pH to 10 with 0.1 N NaOH and filter using a 0.2 μm filter, and 10 ml of amino acids solution was further added to make a final 1 l media.

### Fermentation procedure

A 3.7 l stirred tank reactor (STR) (Bioengineering Co.) with a working volume of 2.5 l was utilized free cells cultivation and SCBBR having same working volume was used for cultivation of immobilized cells. The “Minimal medium” composition as mentioned in section ‘Fermentation medium and cultivation’ was used, and yeast cells of 16 g/l were added in the fermenter in the case of free cells cultivation. Different glucose concentration (50, 100, and 200 g/l) and agitation speed (200, 300 and 500 rpm) were selected to characterize the effect of agitation speed and glucose concentration parameters on the performance of SCBBR regarding mass transfer properties and ethanol productivity.

### Calcium alginate beads preparation and yeast immobilization

During preparation of calcium alginate beads a sterile sodium alginate solution 2.5% (w/v), autoclaved at 121 °C, for 15 min, was prepared in 50 mM phosphate buffer at pH 7. The cell suspension (3%) was mixed with alginate solution for immobilization of baker yeast. In the case of beads preparation, the alginate-yeast solution was drop by drop allowed to dip using 1 ml pipette tip into 200 ml, 180 mM CaCl_2_. Beads were let to harden in this solution for 1 h. Further, beads were rinsed three times with sterile 2% NaCl solution and then with sterile water. The alginate beads with diameters 0.8, 2 and 4 mm were used in experiments. For the preparation of chitosan-coated alginate beads, the above-prepared beads were dipped in sterilized chitosan solution (3% chitosan, 0.1 N HCl, pH 5) for 10 min and later washed 3 times with sterile water.

### Polyethylene sponges immobilization and cultivation conditions

For immobilizing yeast cells, MPEP sponges were initially autoclaved for 15 min at 121 °C and kept overnight at 4 °C to facilitate de-aeration. The MPEP surface was prepared according to the established protocol (Trelles et al. 2010) with some modification. The SCBBR basket was filled with polyethylene sponges, and then pre-cultured yeast cells (16 g/l) were aerobically fermented at 200 rpm and 35 °C for adsorption onto the support. After 2 days, the cell immobilized support was washed with sterile water and later used for an experiment using minimal media.

### Glucose consumption measurements

The DNS method was used for the measurements of immobilized yeast glucose consumption. For each measurement, 0.5 ml sample and 0.5 ml DNS solution were mixed in a 1.5 ml Eppendorf tube, vortex for 10 s, and incubated for 10 min at 90 °C. After incubation, 40% 0.16 ml potassium sodium tartrate was added, mixed by vortex and placed on ice for 3 min. Two hundred microliter of each sample was measured at 575 nm. The obtained results were compared with a calibration curve of different glucose concentration to get actual concentration.

### Ethanol production measurements

The concentration of ethanol produced in a fermentation broth as well as calibration curve was measured with the same method as in previous paper (Hussain et al. 2015a). The fermentation broth samples (each having volume 600 μl) were collected, transferred to an Eppendorf tube and centrifuged at 9000 rpm for 5 min to pellet the cells. Later, 500 μl of the clear supernatant were transferred into a new tube without disturbing the cell pellet, and 5 μl of 1% *n*-butanol was added as an internal standard. After vortex, the samples for 30 s, 1 ml of 25% ethyl acetate was added with a further 5 min vortex. The samples were centrifuged for phase separation, at 5000 rpm and the organic phase was used for gas chromatography (GC). The gas chromatograph equipped with flame ionization detector (FID) was used for sample measurements. The columns used were the 30 and 0.25 mm CP-WAX—57CB (Santa Clara, CA, USA). The column temperature was initially maintained at 120 °C for 2 min, and later the oven temperature was increased at a rate of 10 °C/min until it reached 150 °C. The temperature of injector and detector were kept at 150 and 200 °C, respectively. The flow rate for carrier gas (Helium) was set at 30 ml/min. The injection sample volume was 2 μl. Each experiment was repeated thrice, and the reported value was the mean average.

## Results

### Effect of Stirrer speed and glucose concentration

**Fig. 3:**
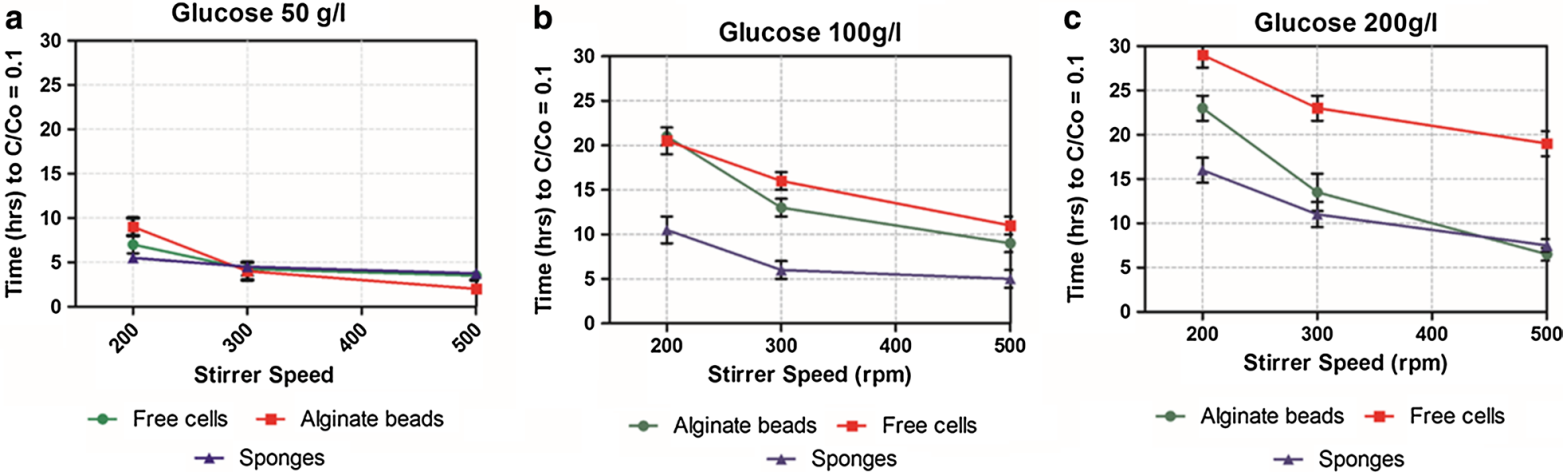
Effect of agitation speed and immobilizing matrices on glucose consumption ** a** at 50 g/l,** b** 100 g/l and** c** 200 g/l

Initial results in Fig. 3a–c shows both free and immobilized yeast cells (alginate beads and sponge, respectively). The result shows how mass transfer properties correlates with the stirrer speed and initial glucose concentration. By varying the stirrer speed from 200 to 500 rpm at a glucose concentration of 50 g/l, the consumption of glucose of up to C/C = 0.1 was observed. For free cells or those immobilized in alginate beads as while as in chemically modified sponges, it can be seen that glucose consumption pattern was more or less the same though the time for consumption of glucose decreases with increase in stirrer speed. When using stirrer speed of 200 rpm, glucose consumption time was nearly 5 h, while at 300 rpm and 500 rpm consumption was significantly lower. The big difference in consumption time when using free and immobilized cells was observed by increasing glucose concentration to 100 and 200 g/l. From Fig. 3b, it can be observed that at 200 rpm with 100 g/l glucose initial amount, sponge immobilized cells consume glucose in 9 h while in alginate beads and free cells consumption was in 19 and 20 h respectively.

By increasing agitation speed to 300 and 500 rpm, glucose consumption time decreases significantly in free cells (15 and 10 h, respectively), alginate beads (12 and 8 h, respectively) and sponge (7 and 6 h, respectively). From these data, we can observe that sponges show no much time difference on both agitation speed as compare to free cells and alginate beads. From these results, we can conclude that the fluid velocity improves the mass transfer of yeast cells immobilized in different matrixes and the magnitude of mass transfer resistance has an inverse relation with stirrer speed. This is because the stirrer speed controls the internal diffusion of the substrate in the case of immobilized cells. The sponge-immobilized cells showed less internal diffusion resistance as compare to alginate beads and have less effect at higher stirrer speed. The results in Fig. 3c support the above observation in which the difference in time of glucose consumption was recorded between free cells (30 h), alginate beads (24 h) and sponges (17 h) at lower stirrer speed (200 rpm) and that tends to decrease (20, 7 and 8 h respectively) at 500 rpm stirrer speed. The higher consumption time in case of free cells might be due to the shear effect of stirrer speed. Moreover, the time difference between sponges and alginate beads might be due to the effect of internal diffusion resistance that can develop concentration gradient inside and on the outer surface of the alginate beads. The concentration gradient is a major problem that can arise in immobilizing technology and it can be improved by using chemically grafted sponges and optimized stirrer speed as shown in above results.

### Effect of immobilization

**Fig. 4:**
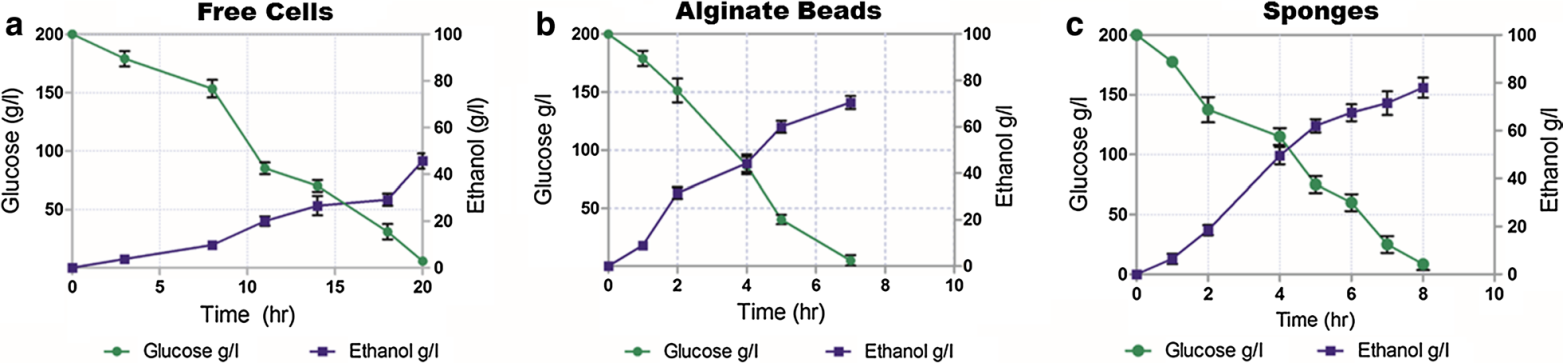
Effect of agitation speed and immobilizing matrix on ethanol yield.** a** Free cells ,** b** Alginate beads,** c** Sponges

By using minimal medium, yeast cells inside the alginate beads were maintained, and its growth was kept at its minimal. Therefore, as the growth increases the concentration of biomass inside the alginate beads increases, and this can enhance the oxygen and nutritional diffusion limitations (Duff and Murray 1988). When using three types of methods for yeast cultivation, the experiments were conducted with free cells in STR, immobilized cells in alginate beads and cells immobilized in sponges in SCBBR with a glucose concentration of 50, 100 and 200 g/l and three different stirrer speeds 200, 300 and 500 rpm. In Figs. 3 and 4, the effect of two important factors, i.e., stirrer speed and immobilizing matrix on ethanol production and ethanol yield are presented. Figure 4a–c shows the initial comparative results of ethanol production between free and immobilized cells using higher glucose concentration of 200 g/l and stirrer speed 500 rpm. Converti et al. (1985) showed in their experiments that higher glucose concentration plays a major role in achieving maximum ethanol productivity (Converti et al. 1985). Ethanol concentration of 85 g/l was obtained when using sponges, while 79 and 60 g/l was obtained for alginate beads and free cells, respectively, at a glucose concentration of 200 g/l (Fig. 4). From these results, it can be concluded that sponges are the best support material for yeast immobilization as can be evidenced by higher ethanol production when compared to both alginate beads as well as free cells.

Another essential part, in these results, is the complete conversion of glucose into ethanol. The higher performance of glucose conversion time was observed in SCBBR than STR reactor. Free cells were observed to take approximately double the time (20 h) to finish one batch process compared to immobilized cells [alginate (7 h) and sponges (8 h)]. With lower stirrer speed, ethanol yield and glucose conversion time were observed as limited by external diffusional resistance. The diffusivity of glucose through boundary layer surrounding the biocatalyst particle plays a major role in achieving maximum ethanol yield that is directly controlled by the structure of immobilizing matrix and stirrer speed of a bioreactor. The medium hydrodynamics in bioreactors exhibit an important influence on glucose conversion and transfer processes (Galaction et al. 2012).

**Fig. 5:**
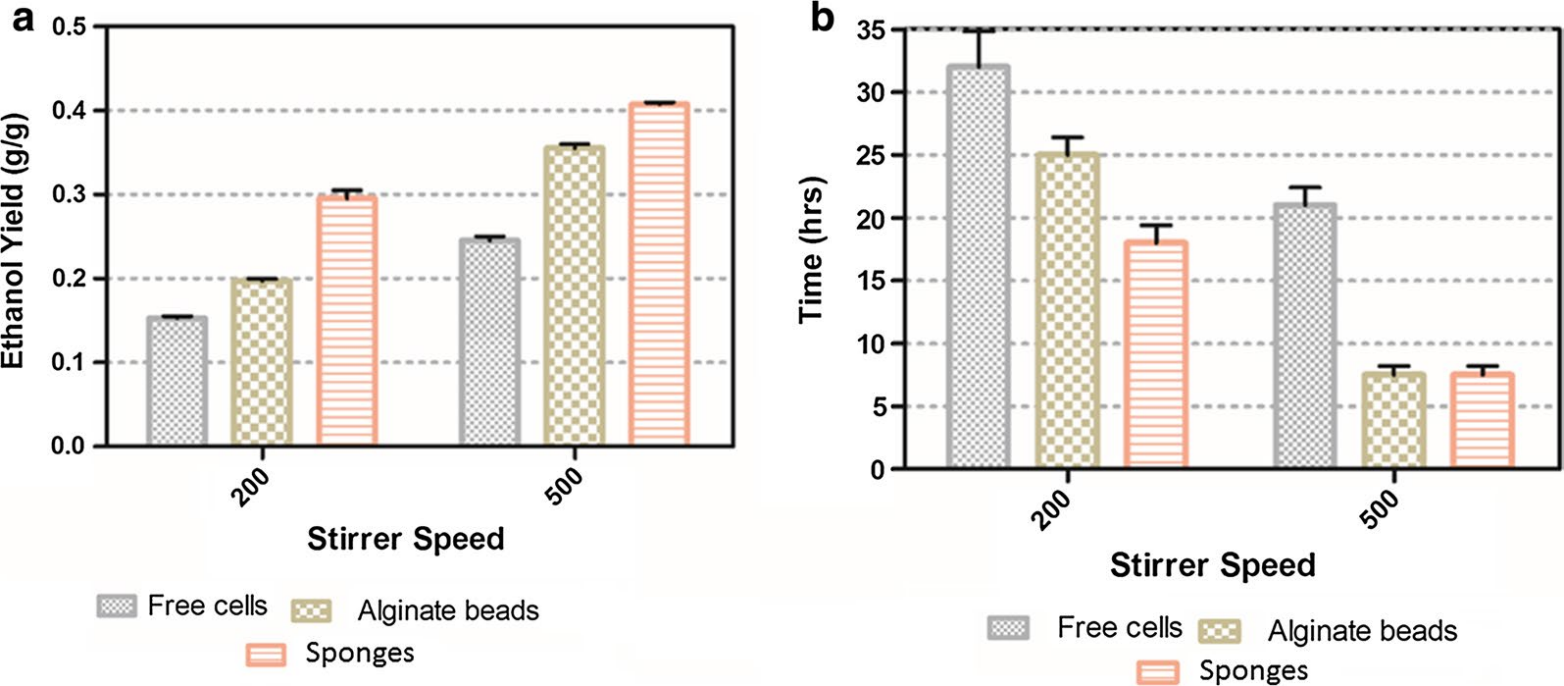
Ethanol yield and glucose consumption time by free and immobilized cells in alginate beads and sponges.** a** Ethanol yield,** b** Glucose consumption time

Figure 5 clearly compares the ethanol yield and time taken for completion of one batch cycle until the level of C/C = 0.1, using a minimum (200 rpm) and maximum (500 rpm) stirrer speed.

While cultivating of free, alginate and sponge immobilized cells at a lower stirrer speed (200 rpm) ethanol yield (0.15, 0.195 and 0.285) was achieved at time (30, 24 and 17 h) and on higher stirrer speed (500 rpm), ethanol yield (0.240, 0.35 and 0.405) increases, respectively. Reduction in glucose consumption time (20, 7, and 8 h) when using free cells in the medium, immobilized cells in alginate beads and sponges was observed, respectively.

The results in Fig. 5 and Table 1 clearly indicate that immobilizing techniques affect the internal diffusion of glucose and consumption rate (Galaction et al. 2010). This offers a more suggestive information regarding the effect of immobilization on ethanol production efficiency, mass transfer phenomena and overall performance of a bioreactor.

**Table 1:** Ethanol yield and glucose consumption time by free and immobilized cells in Alginate beads and sponges

Immobilization method	Stirrer (rpm)	Glucose conc. (g l)	Ethanol productivity (g l^−1^ h)	Ethanol yield glucose (g g^−1^)	Volumetric ethanol yield (g l^−1^)	Glucose consumption time (h)
Free cells	200	200	1.68	0.15	49	29
Alginate	200	200	1.47	0.195	34	23
Sponge	200	200	4.29	0.285	72	17
Free cells	500	200	2.45	0.24	50	20
Alginate	500	200	11.28	0.35	79	7
Sponge	500	200	10.40	0.405	83	8

Ethanol yield (g g^−1^) and Ethanol productivity (g l^−1^ h) refer Eqs. (1) and (2)

1 $$\text{Y}_{(Yield)} = \frac{{P_{1} - P_{0} }}{{S_{0} - S_{1} }}$$

2 $$\text{E}_{(productivity)} = \frac{{P_{1} }}{t}$$

where P_0_ = product at time of fermentation start, P_1_ = product at time of analyzing, S_0_ = substrate at time of fermentation start, S_1_ = substrate left at time of analyzing and t = time in hours.

## Discussion

In biocatalysis, free cells reactions known as a homogeneous catalytic reaction when reactant and catalysts are in the same phase in which mass transfer effect is considered as negligible. When cells are immobilized in different matrices like alginate beads and chemically grafted sponges, the reaction is known as heterogeneous, and reactants and catalysts are in different phases. The catalyst is normally in the solid phase, and reactants are in liquid phase, and the reaction is dependent on the mass transfer (Klaewkla et al. 2011; Hussain et al. 2015b). In this instance, the reaction only takes place when reactants are transferred to the catalytic reaction site by diffusing across external fluid layer around the catalyst (external mass transfer) into pores within the catalyst (Internal mass transfer).

The selection of the best laboratory reactor for intrinsic kinetics investigation is difficult due to transport phenomena that can occur in liquid–solid interfaces. In free cells cultivation, a STR reactor is used due to its advantages of increasing mass transfer rates and well mixing by stirring. The performance of a bioreactor is strongly dependent on these factors: stirrer speed, glucose concentration in the medium and matrixes for immobilizing cells. The performance depends both on the rate of external and internal mass transfer limitations. However, these limitations can also be overcome to a certain extent, by varying the factors mentioned above and its effect can be observed on the performance of SCBBR bioreactor and transport process by consumption behavior of glucose in the medium.

The external mass transfer involves the transport of substrate from bulk medium to the surface of immobilized matrixes. In this case, first resistance issue is the fluid film around matrix surface and its thickness which depends on various physical properties of the fluid, i.e., substrate concentration and velocity of the fluid (8). Higher substrate concentration can raise concentration gradient difference between the bulk liquid and interior surface of beads possibly causing inhibition of substrate. Warnock et al. (2005) stated that in the immobilized cell system, a concentration gradient between bulk and intra-particle medium develop when glucose is consumed, and metabolites are produced (Warnock et al. 2005). It was Talebnia also showed the limitation of substrate transfer to the center of immobilizing matrixes and toxic metabolite out of it (Talebnia and Taherzadeh 2007). It was observed that by using the immobilized system in Packed Bed Bioreactor, lag phase appeared at the start of fermentation due to poor mixing (2), while in SCBBR no lag phase was observed even at higher substrate concentration because of the well-mixing properties of this bioreactor. The internal mass transfer involves transport of substrate from the surface of immobilized matrixes to the site of reaction, and it depends on the properties of matrixes used for immobilizing cells. In our study, two types of matrixes performance were compared, i.e., one was a conventionally used alginate beads, and the other one is newly developed chemically grafted sponges.

Experiments analysis in Fig. 3 shows that glucose consumption time was rather equal at different rotation speed 200, 300 and 500 rpm with lower glucose concentration (50 g/l) using 4 mm size of beads. This indicates that if glucose concentration in the medium is low, a thin film around the matrices develops and there might be a linear substrate gradient across the thin film.

The difference in time in glucose consumption between free and immobilized cells (alginate and sponges) tends to increase by increasing the glucose concentration (100 and 200 g/l) in the medium and this might be due to a thick layer developed around the particle which can increase the concentration gradient in and outside the particle. The substrate is not equally available because substrate could not reach the middle of the particle. Therefore, this area is deprived of the substrate, and that can have an effect on the whole productivity of the system. This can also be observed when using alginate beads and not with sponges as sponges take less time to consume glucose and might have no external film developed because of bigger pore size that enhances the intra-particle flow (Warnock et al. 2005). It can also be concluded that by using sponges we can eliminate external and internal mass transfer limitations of substrate concentration gradient. The same behavior was observed by Galaction et al. (2012) with alginate beads in the bioreactor when using higher glucose concentration (150 g/l), glucose concentration on the surface of the beads increases and glucose consumption rate was reduced due to substrate concentration gradient (Galaction et al. 2012).

To overcome all this, there is one major factor which can help eliminate the mass transfer limitations i.e. stirrer speed. The stirrer speed helps to maintain the intra-particle flow by maintaining total flow rate and to the extent that no shear effect exerts on cells performance as the cells can suffer internal damage during higher stirrer speed that could be attributed to a weaker cell membrane. The poor velocity also can create problems of the concentration gradient, bridging, and channeling, especially when using alginate beads. The ideal flow pattern in any reactor is not always possible, but we can improve and avoid these problems by recognizing optimum factors. The optimized stirrer speed or efficient mixing in a bioreactor has importance in immobilized cell system as it ensures optimal temperature and concentration gradients at the catalyst surface and all heterogeneous catalysis depend on these transport processes since it helps in elimination of CO_2_ and ethanol (Armando Gamarra et al. 1986). The results can be supported by Converti et al. 1985 work where they studied the effect of stirrer speed on metabolic activities of *Saccharomyces cerevisiae* and found that stirrer speed is a major factor that affects the product yield (Converti et al. 1985).

Figure 3 indicate an effect of stirrer speed on free and immobilized cells. Glucose consumption time was higher at lower stirrer speed (200 rpm) both free and immobilized cells due to inefficient mass transfer. To make it efficient substrate should reach the yeast cells by diffusion and convection through the external liquid film, liquid–solid interface, and resistance caused by liquid and micro-colonies of yeast within the particle. The stirrer speed alters the diffusion and convection process of the substrate. If stirrer speed is not enough, the substrate concentration on the surface and within the matrix will be lower than the concentration in the bulk medium. In this case, in the center of the matrix, a dead necrotic zone can develop, and only cells biomass around the periphery of the matrix will consume substrate. These reasons support our result that at lower stirrer speed cells take a longer time to consume glucose. Yeast cell viability and metabolism is also severely affected by mass transfer properties. Therefore, glucose and other nutrients in fermentation medium must diffuse to the yeast cells and product or other metabolites should diffuse out into the medium otherwise these could be toxic or show inhibition to cells and can decrease cell productivity (Pilkington et al. 1998). While increasing the stirrer speed to 300 rpm and 500 rpm, glucose consumption time tends to decrease at all glucose concentration (50, 100, and 200 g/l). The results clearly indicate that there is less concentration gradient, as substrate traveled easily to the reaction site of yeast cells and is consumed as faster as it is provided by the diffusion process. On the other hand, the shear effect of higher stirrer speed (even at 500 rpm) has been observed in Fig. 3c indicating that the time for glucose consumption is higher in the case of free cells as compare to alginate beads and sponges. The higher consumption time in case of free cells confirmed the shear effect of stirrer speed as well as the glucose. Mechanical stress (shear stress) is one of the factors having an impact on yeast cell wall and its functionality like reduction in viability and vitality. In traditional ethanol fermentation technology, freely suspended yeast cells were used in the batch. Moreover, several drawbacks are found in this technology like more fermentation time and low volumetric productivity, due to continuous changes in the external physical factors and biological activity of yeast. To achieve optimal conditions for metabolic activity, yeast must maintain the intracellular physical and chemical parameters (Bauer and Pretorius).

In our previous study (Hussain et al. 2015a) using packed bed bioreactor, we found that at the start of fermentation process lag phase (an adaptation phase) time is more at a lower flow rate and less at higher flow rate. The presence of lag phase is the indication of concentration gradient around the surface and within the beads that can be controlled by the optimized flow of the fluid medium. In SCBBR, no lag phase was found due to efficient mixing or mass transfer and less concentration gradient to such an extent to induce the lag phase. In SCBBR pH and temperature are controlled by complete mixing with the help of agitation. Consequently, the hydrodynamics of the broth in and around the basket shows an important effect on the mass transfer processes involved in substrate conversion. The basket has the advantage of permitting greater contact between reactants and biocatalyst, which in turn increases the reaction rate and efficiency of bio-catalytic reaction and the bio-catalyst is separated from the reaction mixture simply by draining the circulating liquid (Baltaru et al. 2009).

The SCBBR produces higher ethanol than the STR used as a control. In Fig. 4c increasing stirrer speed (500 rpm) using higher glucose concentration (200 g/l), ethanol volumetric productivity was increased significantly in the sponge (85 g/l) as compared to alginate beads (79 g/l) and free cells (60 g/l). It can be concluded that sponges are the best support material for attaining higher ethanol productivity. Cell immobilization has commonly been used to improve the performance, cell physiology, and economics of most fermentation processes. However, for immobilized cells, care must be taken to ensure that the support is not damaged and the yeast cells do not suffer from shear stress as it has been observed in free cells.

Figure 4a–c indicate that immobilized cells preserved their activity than free cells and are more resistant to heat and shear effect. This might be because free cells are taking double time (20 h) to complete one batch process than support materials [alginate beads (7 h) and sponges (8 h)] as this can be attributed to the inhibition and severe shear effect of stirrer speed on free cells. Free cells are more sensitive to nutrient depletion, pH variations, and certain inhibitory compounds because these are directly exposed to the changing environment. For maintaining metabolic activity, several mechanisms exist in all unicellular organisms that let them observe and adopt the environmental change, and if they fail to adapt these changes, then it leads to several problems like reduced growth rate, cell death, inhibition and concentration gradient (in heterogeneous reactions) (Bauer and Pretorius). Bleoanca and Bahrim (2013) also described yeast stress factors that can directly affect cellular activity and overall fermentation performance. While in the case of immobilized cells stirrer speed controls the diffusion of substrate and concentration gradient. The concentration gradient is the difference of (substrate or product) concentration between two phases (Bleoanca and Bahrim 2013). The external concentration gradient is the difference in concentration between the bulk liquid and external surface of the beads (Salmon and Robertson 1987).

The effect of higher glucose concentration on ethanol productivity can be observed in Fig. 4a–c, and it indicates the reduction in ethanol productivity in alginate beads as compare to sponges. This could be the inhibition or reverse of reaction due to a higher rate of reaction upon increasing substrate concentration. Also, (Nikolić et al. 2009) observed a significant decrease in ethanol yield on the addition of sugar concentration in fermentation medium and (Galaction et al. 2010) found intra-phase resistance induces the substrate inhibition which is directly related to the glucose concentration gradient (Nikolić et al. 2009; Galaction et al. 2010). The lower ethanol yield obtained when using free cells (Figs. 4, 5) might be due to the effect of glucose inhibition that tends to increase upon using higher glucose concentration. The efficiency and physiology of free cells are markedly affected by the use of higher glucose concentration inhibition and the shear effect of higher stirrer speed. As the cells can exhibit different metabolisms which depend upon their microenvironment and reactor operating conditions (Roberts and Fisher 2000). Inhibition of yeast growth and metabolic activities by high initial substrate concentration was also observed by Lee et al. (2012), while (Galaction et al. 2010) showed results depicting that substrate or product inhibition phenomenon could limit the efficiency of ethanol production (Lee et al. 2012; Galaction et al. 2010). Moreover, the productivity difference between sponges and alginate beads shows the presence of internal diffusional resistance that can create a concentration gradient.

Figure 5 shows that there is no significant time difference for consumption of glucose observed between sponges and alginate beads at higher stirrer speed (500 rpm). The reason might be due to the removal of diffusional limitations in and around the supporting materials. From this, it can be concluded that the magnitude of resistance to the internal diffusion is directly related to the types of immobilizing matrixes and also on glucose concentration gradient (Galaction et al. 2010; Engasser and Horvath 1973). The consumption time of glucose, i.e., traveling of the substrate from the outer surface to inside the matrix depends upon the stirrer speed and texture of the matrix used for immobilizing cells. The results in Fig. 5 indicate that the sponges take less time to complete one batch process than alginate beads and free cells. This might be because cells immobilized in sponges have very less mass transfer limitations and no diffusional barrier by immobilizing reagent as compared to alginate beads. Although alginate beads are porous, a further disadvantage regarding internal mass transfer is that they do not have convective flow inside and nutrients traveled to the cells only by diffusion (Najafpour et al. 2004; Shafaghat et al. 2011). We observed that a SCBBR with yeast cells immobilized in polyethylene sponge gives higher ethanol production at a higher glucose consumption rate, and this productivity is due to higher mixing efficiency and reduced external as well as internal mass transfer limitations. In the near future, we will focus more on improving the process efficiency and more successive fermentations will be conducted to demonstrate the stability of the immobilization.

## Abbreviations

SCBBR: Stirred-Catalytic-Basket-BioReactor
GC: gas chromatography
